# Father wellbeing: self-efficacy, positive engagement, and reading with children

**DOI:** 10.3389/fpsyg.2026.1730393

**Published:** 2026-03-04

**Authors:** Claudine Habak, Anna Marie Dillon, Kay Gallagher, Sumaya Saqr

**Affiliations:** 1 Cognitive Neuroimaging Unit, Emirates College for Advanced Education, Abu Dhabi, United Arab Emirates; 2 Language Cognition and Growth, Emirates College for Advanced Education, Abu Dhabi, United Arab Emirates; 3 Faculty of Education, University of Ljubljana, Ljubljana, Slovenia

**Keywords:** father wellbeing, wellbeing scale life and work, parenting self-efficacy, positive engagement with child, home reading, multicultural, social support, child literacy development

## Abstract

**Introduction:**

Father involvement benefits the development and wellbeing of children, and yet, the wellbeing of fathers themselves is rarely considered, as is its relationship with positive engagement activities. This work addresses the wellbeing of fathers through basic psychological need satisfaction, and the links of wellbeing with fathering self-efficacy (father parenting confidence) and with positive engagement with children through father-child home reading.

**Methods:**

Responses to an online questionnaire consisting of the validated Fathering Self-Efficacy Scale (FSES), the developed Being Well in Life and Work scale (BWLW), a scale of reading beliefs and a scale of engagement in literacy development with children, were completed by 122 fathers of diverse cultures and languages in a multinational cosmopolitan setting.

**Results:**

Fathering self-efficacy was associated significantly with father wellbeing through the self-efficacy aspects of positive engagement and family provision, and the sense of family provision was associated with engagement in literacy activities. Wellbeing levels were relatively high at 75% of the scale. Fathers reported infrequent and widely ranging engagement with children in literacy activities, but frequent engagement in play.

**Discussion:**

Findings suggest that 1) opportunities for father self-efficacy through social connectedness with peers and positive engagement can support the wellbeing of fathers, and 2) that fostering father self-efficacy in family provision, along with father-targeted resources and approaches (such as incorporating play), can support father engagement in their children’s literacy development.

## 1 Introduction

The positive role of father involvement in child development has been well-established, with contributions to child and family health (Allport et al., 2018; Yogman et al., 2016), to children’s academic growth (Whitney et al., 2018), and to children’s wellbeing (Kesebonye and Amone-P’Olak, 2021; Mangiavacchi et al., 2021). Father involvement in positive engagement activities with children is supported by self-efficacy or confidence in one’s ability to parent (Bouchard et al., 2007; Sevigny et al., 2016; Fortin, 2023). Yet, little work addresses the wellbeing of fathers themselves, or its relationship with positive engagement activities associated with self-efficacy, that are beneficial to the father and to the child. One notable such father-child engagement activity is interactive shared book reading for pleasure, which supports young children’s literacy development while offering opportunities for positive father-child engagement (Gallagher et al., 2025). When shared parent–child reading is construed as a meaning-focused activity, rather than a literacy teaching activity, fathers who read picture story books with their young children at home report feelings of joy (Swain et al., 2017; Dillon et al., 2024) and closeness to their children (Gallagher et al., 2025).

Importantly, parental reading with young children has been found to have a positive effect on their educational attainment, and in particular, children whose fathers read regularly with them have higher attainment in reading at age five than other children (Norman and Davies, 2023). A review of recent studies into father-child interaction patterns during shared reading reports that fathers tend to elicit higher quality language from their children than mothers do (Cutler and Palkovitz, 2020). Overall, parental engagement in shared book reading supports children’s oral language development (Wang et al., 2023), their development as readers (Cutler and Palkovitz, 2020; McWayne et al., 2013; Ortiz, 2000; Varghese and Wachen, 2016), their socio-emotional growth (Cutler and Palkovitz, 2020; Schapira and Aram, 2020), and their long-term cognitive function and wellbeing (Sun et al., 2024). Concurrently, fathering self-efficacy (Bandura, 1982) or parenting competence – confidence in one’s ability to parent, is associated with the wellbeing of fathers, by satisfying the psychological needs for fathering and with increased engagement with children (Bouchard et al., 2007; Fortin, 2023). However, limited work has addressed these connections, and this paper explores the links between father wellbeing, fathering self-efficacy, and father engagement in shared reading with their children at home.

### 1.1 Father wellbeing and interactions with children

The wellbeing of fathers is usually addressed in terms of the influence of father involvement on the wellbeing of the child or family (Allport et al., 2018; Sarkadi et al., 2008), or around perinatal depression in fathers when transitioning to parenthood (Garfield et al., 2014; Ndzi and Holmes, 2024; Younis et al., 2024). Earlier studies comparing the wellbeing of fathers and mothers addressed wellbeing through depression, physical health, and self-esteem, and its associations with time spent with children and household chores (e.g., Blair and Hardesty, 1994). The conceptualization of fathering has since become more sophisticated, going beyond time spent with children or in the household, to encompass affective, cognitive and behavioral facets, along with the quality of father-child relationships (Cabrera et al., 2018; Lamb et al., 1987; Lamb and Oppenheim, 1989; Palkovitz, 2019; Pleck, 2010), and the wider socio-cultural context (Volling and Palkovitz, 2021). These aspects are reflected in fathers reading with their children, which constitutes a positive engagement activity, where, for example, the quality of father-child language exchange is more important than its quantity (Rowe et al., 2017), and father-child reading offers a space for choices, affective connection, and cognitive exchange (Cutler, 2023).

Parental wellbeing can be mediated by positive aspects, such as purpose in life, psychological needs, and positive affect, and by negative aspects, such as strained finances, partner relationships, and negative affect (Nelson et al., 2014). These influences can vary between fathers and mothers, with fathers experiencing greater life satisfaction, happiness, positive affect, and meaning, along with less depression than do mothers (Nelson-Coffey et al., 2019) or non-father men (Nelson et al., 2013); these maternal-paternal patterns are beyond the present scope, but they are introduced to highlight the importance of addressing fathers specifically. Surprisingly little evidence exists on the holistic, non-clinical wellbeing of fathers themselves, with much of the work focusing on the relationship between wellbeing and involvement. For example, increased father involvement is linked with the psychosocial health of fathers (and of mothers and children), including depressive symptoms, personal wellbeing, and self-efficacy (Mallette et al., 2021). It is also associated with fathers’ affective/emotional communication while playing with their children (Lewis et al., 2009), with higher parenting competence (which is closely related to self-efficacy), and with feelings of closeness with their children (Ehrenberg et al., 2001). Taken together, this suggests that wellbeing and self-efficacy interact in shaping father-child interaction and engagement.

### 1.2 Wellbeing, psychological needs, and fathering self-efficacy

Self-efficacy, or confidence in one’s ability to carry out an action (Bandura, 1982) such as parenting, consists of feeling a general sense of competence in parenting across a range of beliefs: for example, feeling able to engage in specific activities like reading, feeling competent in parenting roles such as teaching something, playing with, or loving and supporting one’s child. Fathering self-efficacy can support wellbeing through mechanisms such as parenting stress reduction (Schoppe-Sullivan et al., 2021; Sevigny and Loutzenhiser, 2010), social support for new fathers (Gaillot and Wendland, 2024), and family climate (Bandura et al., 2011). In addition, father-targeted approaches can support key aspects of fathering. For example, fathers of children in neonatal intensive care provided with knowledge and skills by nurses showed improved parenting confidence and knowledge during the hospital stay, and more importantly, at 4–5-year follow-up (Hearn et al., 2020), which included increased involvement and feelings of closeness (Clarkson and Hearn, 2021). Furthermore, when father-specific information and resources are lacking, father self-efficacy decreases (Gaynor et al., 2024).

These elements of psychosocial and affective engagement, along with parenting competence, connect with the three core psychological needs that support positive functioning across aspects of wellbeing and across cultures (e.g., Chen et al., 2015; Disabato et al., 2016; Martela and Sheldon, 2019). Basic psychological needs defined within self-determination theory constitute the extent to which individuals feel that the three basic psychological needs are satisfied: a sense of competence or feeling capable, a sense of relatedness or feeling close or connected with others (belonging), and a sense of autonomy or feeling in charge of or responsible for one’s own actions (Deci and Ryan, 2000, 1985; Ryan and Deci, 2017, 2000). In the daily life of fathers as individuals, these components are manifest in feeling capable of undertaking activities in life and work, such as taking care of responsibilities or knowing how to interact with a child (competence); a sense of care and feeling connected with others (belonging); and a feeling of having choices, such as life-work balance or choosing activities (autonomy).

### 1.3 This study

In light of the foregoing, the aim of this study is to address children’s literacy with fathers, along with father wellbeing and self-efficacy, to guide father-specific information for literacy development with their children, and support key affective, cognitive, behavioral, and relationship aspects of fathering. Based on the existing literature, we hypothesized that (a) fathering self-efficacy would be associated with wellbeing, and (b) that fathering self-efficacy and wellbeing would be associated with positive beliefs about literacy and with positive engagement in literacy activities.

The present work reports on the wellbeing and self-efficacy dimensions of a father-child home reading study carried out with culturally and linguistically diverse families with at least one child aged 4–6 years in Abu Dhabi. Furthermore, while there are numerous wellbeing tools, one key element of the present study was to address the wellbeing of the individual in the context of aspects of life and work relevant to fathers as individuals; a subgoal was to test a wellbeing scale applicable to fathers. To preempt the results, we find that fathering self-efficacy is associated with father wellbeing. Furthermore, fathering self-efficacy is associated with engagement in activities that support literacy development, but not with beliefs about literacy.

## 2 Materials and methods

The portion of the study presented here reports on father perceptions through an online self-report survey questionnaire administered via Qualtrics.^1^

### 2.1 Participants

Individuals identifying as fathers of a child/children aged 6 years or under living in Abu Dhabi, a multicultural and multilingual city in the Gulf region (*N* = 225), accessed the questionnaire, and 162 responded.

#### 2.1.1 Demographics

After response examination and data cleaning, the sample comprised 122 participants from 20 nationalities across West Asia (including the UAE), Africa, Europe, the Indian subcontinent, Australasia, and North America. The mean age was 39.4 ± 6.2 years (range 26–64 years). In terms of employment, 90.6% were employed full-time, 3.4% part-time, and 6.0% were not employed. Occupations encompassed professionals in general (63.1%), civil service (23%), while 0.8% were retired, and 13.1% did not specify their occupation. Education levels were relatively high with 67.2% holding university degrees but extended over a broad range, with a distribution consisting of: below high school (0.8%), High school or equivalent (20.5%), Certificate or Diploma (7.4%), Professional Qualification (4.1%), bachelor’s degree (38.5%), and graduate degree (28.7%). Participants reported a range of family sizes, from 1 to 12 children, with the median family size being 3 children (30.8%), followed by 2 children (22.5%), and 1 child and 4 children (10.0% each).

### 2.2 Ethics

The study was carried out in accordance with the WHO’s Declaration of Helsinki with informed consent, and was approved by the Institutional Review Board of Emirates College for Advanced Education (RP-209-2023, 25 February 2023). Because this portion of the study was carried out anonymously, no identifiers were collected (names, IP addresses, etc.), and informed consent consisted of participants selecting the option to participate (or not) after being presented with the study information. In addition, participants could exit the survey at any time and skip any questions they chose. Potential participants were invited to contact the research team with any questions prior to proceeding, in which case the research team could identify a potential participant. However, the team had no way of knowing whether the individual had chosen to proceed. Invitations containing the survey link and brief information about the study were sent from the institution’s Research Office to the university community and affiliated communities, along with wider advertisement on community groups. Sending the ad from a university could bias the sample toward more educated participants, however, only one participant reported being a professor, and most professions were not associated with university roles.

### 2.3 Questionnaires

The questionnaire consisted of eight main sections and was administered online via Qualtrics. Questions appeared in English and in Arabic. The 8 sections consisted of (1) fathers’ demographic information (10 questions), including age, nationality, education, employment status, occupation, number of children with their ages and gender, time spent interacting with children on weekdays and weekends separately, plus an open-ended questions on activities carried out with children; (2) Home and father-child language practices (three questions); (3) Beliefs about children’s reading (nine questions on a 4 point Likert Disagree-Agree, plus “I don’t know”); (4) Reasons for reading with their children (11 options); (5) Barriers to reading with their children (15 option selections, including “other”); (6) Frequency of positive engagement activities known to support language and literacy-development carried out with their children (six questions on a 5-point Likert (Rarely, A few times per month, about once a week, several times per week, daily) and enjoyment of reading (two questions on a 5-point Likert (try to avoid it, do not enjoy it, enjoy it, enjoy it very much); (7) Wellbeing across Life and Work (26 questions on a 5 point Likert Almost none of the time – Almost always); and (8) Fathering self-efficacy (20-questions, 5 point Likert Strongly disagree – Strongly agree).

Consistent with the purpose of this study, the present work reports on the sample demographics and survey items related to father wellbeing and its associations with self-efficacy, beliefs about children’s literacy and positive engagement in literacy-supporting practices. In the context of children’s literacy and language development supported by father-child shared reading, where a sample of fathers participated in workshops on reading with children, mixed-methods findings addressing father-child home language practices, with reasons for reading, and barriers to reading are published elsewhere (Gallagher et al., 2025).

#### 2.3.1 Fathering self-efficacy

Because of the relationship between fathering self-efficacy and the satisfaction of psychological needs (wellbeing) and involvement with children, fathering self-efficacy was assessed using the Fathering Sel-Efficacy Scale – FSES (Sevigny et al., 2016). The scale comprises 20 questions across three subscales: Positive Engagement – teaching, understanding, and responsive interaction with child/ren (12 questions), Direct Care – tending to child/ren (4 questions), and Financial Responsibility – providing for the family (4 questions). The FSES was designed with response options on a 9-point Likert scale ranging from completely disagree to completely agree, and following approval from the FSES corresponding author, it was modified to a 5-point scale with anchors of strongly disagree to strongly agree. The FSES was developed with fathers with at least one child aged 2–6 years regardless of relationship status (Sevigny et al., 2016); the present study included fathers with at least one child aged 4–6 regardless of relationship status, so the FSES was selected for its fit to the demographics, and for the items’ relevance to the diverse cultural context of the present study.

#### 2.3.2 Wellbeing scale

With our focus on the wellbeing of fathers and the related area of fathering self-efficacy (assessed using the FSES), the wellbeing questionnaire was developed to address the person in context, rather than specifically as a father, thereby complementing the FSES. Wellbeing was operationalized through the three psychological needs, with items incorporating feelings of competence, autonomy, and relatedness (Deci and Ryan, 1985; Ryan and Deci, 2000, 2017) along dimensions of life satisfaction with affective-cognitive tone (Diener et al., 2018, 1999) across life, home, and work. Aspects pertaining to perceptions of self-wellness and life were addressed, along with work and social relationships. The inclusion of work satisfaction was consistent with the importance of indirect care, such as financial responsibility to fathers (Pleck, 2010; Sevigny et al., 2016). Analogously, social support is beneficial to father involvement (Fortin, 2023) and to wellbeing, and is addressed here for the individual through their sense of social connection. Specifically, items and their foundation are shown Appendix Table 1, where 26 items incorporated the 3 pillars of psychological needs with respect to perceptions of the self (e.g., “I am happy,” “I easily get stressed,” “I have enough energy,” “I am comfortable with my life as it is,” “I like facing challenges”; 9 items), to aspects of life interaction (e.g., “I have enough time for myself”; 5 items), to outlook towards work (e.g., “I look forward to going to work,” “I have a feeling of belonging at work”; 5 items), and to social relationships and closeness (“I feel connected to my friends,” “I enjoy being with others at work,” “I feel close to my family”; 7 items).

Even though numerous validated scales of wellbeing exist, most address only partially the relational components identified as important to fathers, or focus only on life or work, but rarely both. For example, the Basic Psychological Need Satisfaction and Frustration Scale (Chen et al., 2015) and its work counterpart (Olafsen et al., 2021) each comprise 24 items. Another example is the Work-related Basic Need Satisfaction scale (Van Den Broeck et al., 2010), which would need to be complemented by a scale addressing aspects of life. Using any of these or other pre-validated scales would have significantly lengthened the questionnaire, leading to more incomplete responses due to survey length. Accordingly, as described above, a relatively brief questionnaire was developed to address psychological needs satisfaction across both life and work.

All items were translated to Arabic, back-translated, then revised by a native Arabic speaker fluent in English. The initial survey distribution plan had participants selecting their preferred language. Still, after piloting the survey with a small number of Arabic-speaking Emirati fathers, they preferred to view questions in Arabic and English simultaneously, so this was implemented prior to survey distribution. In addition, these fathers provided feedback on non-validated items and adjustments were made accordingly.

### 2.4 Data analysis

Responses were examined, and of the initial responses, 122 participants completed all sections relevant to this study (the latter portions of the survey). The other participants responded to earlier portions of the questionnaire but stopped responding after one of the literacy-related sections (i.e., sections 3, 4, 5, 6). For the 122 participants, there was no consistent pattern of unanswered questions, other than two participants not responding to questions regarding literacy-supporting activities (section 6; one did not respond to any questions - questionnaire completion of 86%, and the other responded partially, with questionnaire completion of 90%). Three additional participants did not respond to the last two Direct Care questions or the Financial Responsibility questions of the FSES, which also constituted the last 6 questions of the questionnaire (89% questionnaire completion). Two participants completed 1/3 of the wellbeing items (different portions, 75% completion). All others responded to 94% or more of the questions, with more than half (63%) of the participants responding to all questions. Two questions pertaining to time spent interacting with children per day on (1) weekdays and on (2) weekends, seem to have been interpreted differently: many responded per day (e.g., 2 h), but others seem to have responded for the week/weekend overall (e.g., 30 h). Accordingly, values beyond 8 h per day were removed, but this eliminated almost half the responses, so time with children was not included in the analyses. The data for reading beliefs, literacy activities, the FSES, and the wellbeing scale (Being Well in Life and Work) did not meet assumptions of normality; appropriate tests and corrections were used and described below.

Analyses were carried out using Jamovi.^2^ For each of the scales, reliability analyses for each section/scale using Cronbach’s *α* were carried out, along with descriptive statistics. Within a given scale, repeated measures ANOVAs were run to compare levels across subscales; where assumptions were violated, a correction was used on the effects (Greenhouse–Geisser or Huynh-Feldt, according to the test value), and a Bonferroni correction on the post-hoc comparisons. The structural validity of the wellbeing scale in the present context was evaluated using Exploratory Factor Analysis (EFA with Maximum Likelihood extraction and Oblimin rotation), internal consistency measures, and descriptive scale analyses. Convergent validity was tested by running Pearson correlations between the wellbeing scale and the FSES. To ascertain that all items contributed sufficiently to the literacy scales in this context, confirmatory factor analyses (CFAs) were run for each.

To assess father involvement in positive engagement, two generalized linear models were run to test the contribution of self-efficacy and wellbeing (subscales as independent variables) to the overall score of the positive engagement literacy activities, and also to the item regarding reading paper books, as it is a highly pertinent marker of engagement with children for language and literacy development. To assess the contribution of father self-efficacy to father wellbeing, a generalized linear model was run with the self-efficacy subscales as predictors of wellbeing. Generalized linear models were selected over regression models because the residuals were not normally distributed (*p* < 0.05 on the Shapiro–Wilk and Anderson-Darling tests), except for self-efficacy and literacy engagement.

## 3 Results

Below, is the structure of the wellbeing scale (Being Well in Life and Work - BWLW) in the fathering context of this study, followed by the results for each scale, and finally, the results of the generalized linear models to address the contributions of self-efficacy and wellbeing in father-child shared reading engagement.

### 3.1 Engagement in literacy development activities

**Figure 1:**
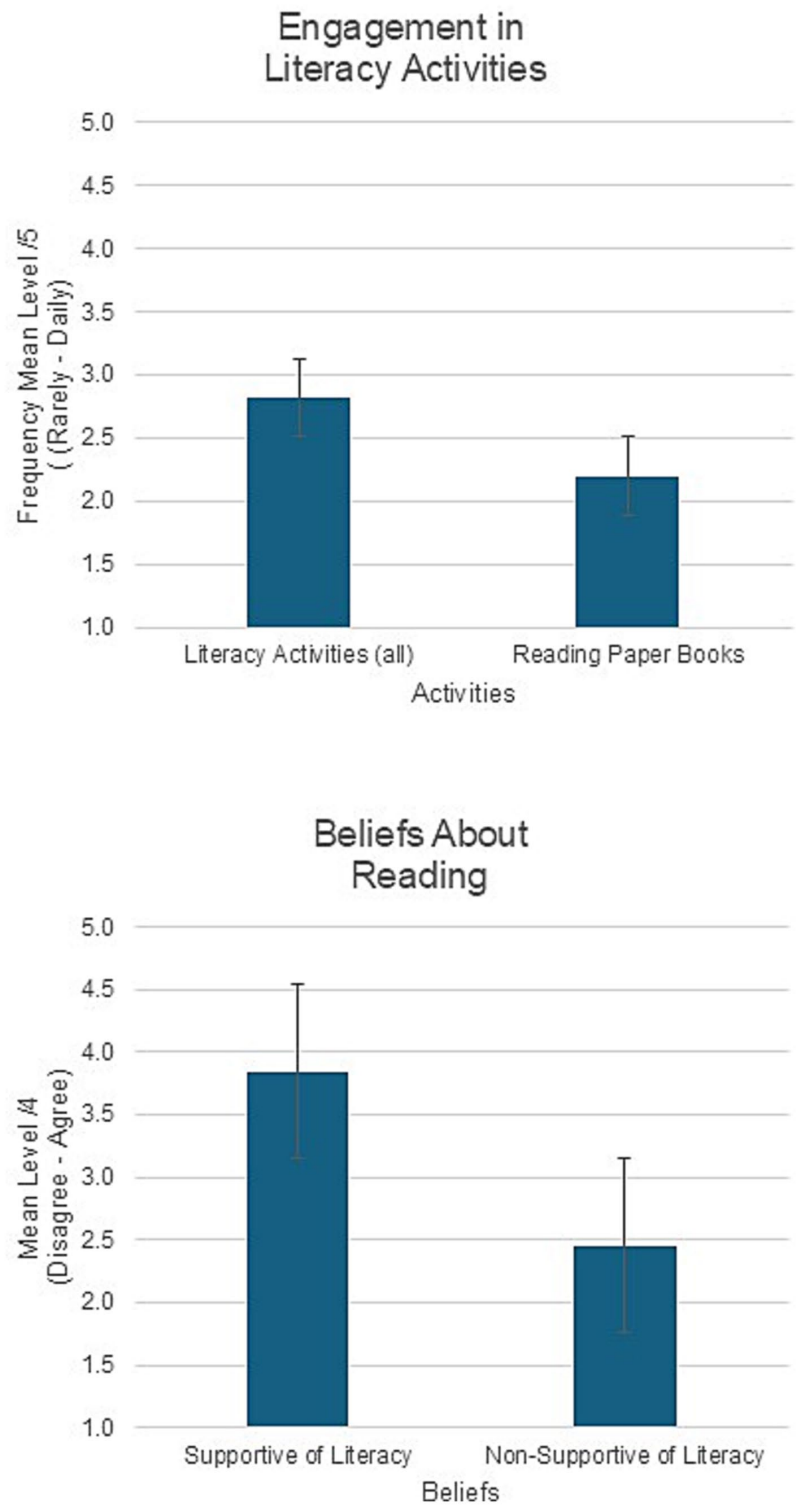
Engagement in literacy activities and beliefs about reading. Means and standard error of the mean.

As the study focuses on the positive engagement of fathers in children’s literacy development and its association with father wellbeing and self-efficacy, results of the positive engagement activities that support literacy are addressed here through CFA (item loading threshold of 0.5). Of the 8 items in this scale, 5 loaded, yielding good reliability (Cronbach’s *α* = 0.80), so an overall mean score was based on these 5 items. Items and their loadings are shown in Table 1. The items that were removed for weak loadings, consisted of frequency of play (0.45), reading e-books or magazines (0.38), and enjoyment of reading with one’s child (0.42). The items, their means, and loadings are shown in Table 1, along with scale reliability. Mean frequency of engagement in activities that support literacy development (Figure 1A) was close to once a week at 2.82 ± 0.087, and reading paper books or magazines was slightly more frequent than a few times per month at 2.64 ± 0.11, with a wide range of frequencies.

**Table 1:** Engagement in activities that support literacy development.

Engagement in literacy development activities (*α* = 0.80)	Mean	SD	Item loading
Composite based on loading items	2.82	0.96	NA
How often do you tell stories out loud with your child who is who is under the age of 6? - this does not include reading	2.76	1.24	0.83
How often do you read paper books or magazines with your child who is under the age of 6?	2.64	1.36	0.78
How often does your child (who is under 6) ask to be read to?	2.98	1.38	0.71
How often do you sing or recite rhymes to your child who is under the age of 6?	2.50	1.43	0.57
How much does your child who is under the age of 6 typically enjoy reading with you or looking at books or magazines together?	3.24	0.81	0.52
How often do you play with your child who is under the age of 6?	4.14	0.92	—
How often do you read books or magazines with your child (who is under 6) using electronic devices?	2.20	1.22	—
How much do you typically enjoy reading with your child (who is under 6) or looking at books or magazines together?	3.26	0.65	—

Items and their loadings.

### 3.2 Beliefs about Reading

Parental beliefs about reading can be related to their engagement in literacy activities, and questions about beliefs were categorized as either supporting literacy and language development or not. Of the nine questions, eight loaded onto the model, with five reflecting supportive beliefs (used as a subscale score), and three non-supportive beliefs (used as a second subscale score). The item pertaining to reading favorites over and over did not load. Item categories, items, their means and loadings are shown in Table 2, along with scale reliability based on Cronbach’s *α*. The subscale of beliefs supportive of literacy yielded good reliability (*α* = 0.81), whereas the subscale of non-supportive beliefs did not (*α* = 0.58), and was not used in further analyses. Mean strength of beliefs about reading (Figure 1B) that are supportive of language and literacy development was near “agree” at 3.85 ± 0.09, and strength of beliefs that are not supportive were centered between “disagree a little” and “agree a little” at 2.46 ± 0.08. In addition, there was one significant but very weak (0.18) correlation with one aspect of wellbeing (Self) and is therefore not considered, and no significant correlations with any other aspect of wellbeing, self-efficacy, or with the overall engagement in literacy-development activities.

**Table 2:** Beliefs about literacy that are supportive or non-supportive of literacy development.

Beliefs about literacy	Mean	SD	Item Loading
Supportive of literacy development (*α* = 0.81)	3.89	0.38	NA
It is good to allow your child under the age of 6 to “read” familiar books by retelling the story from memory using the pictures	3.82	0.50	0.85
It is good to point to the print as you read with a child under the age of 6	3.86	0.42	0.75
It is good to encourage a child under the age of 6 to discuss what is being read	3.91	0.36	0.72
Parents should ask questions at the end of a story in order to check their children’s understanding	3.79	0.55	0.69
It is important that children under the age of 6 see their parents reading	3.85	0.45	0.67
It is good for a child under 6 to hear favorite stories read over and over again	3.70	0.67	—
Non-supportive of literacy development (*α* = 0.58)	2.56	0.92	NA
Schools should be primarily responsible for teaching children under the age of 6 to learn to read	2.93	1.14	0.70
Children should be a certain age before they can begin to learn to read	2.39	1.14	0.54
When reading a book to a child under 6, you should not encourage them to join in because it’s better if they listen without interrupting	1.98	1.16	0.50

Means and standard deviations are shown for each item and composite subscale scores. Scale reliability is indicated by the Cronbach’s *α* value.

### 3.3 Self-efficacy

**Figure 2:**
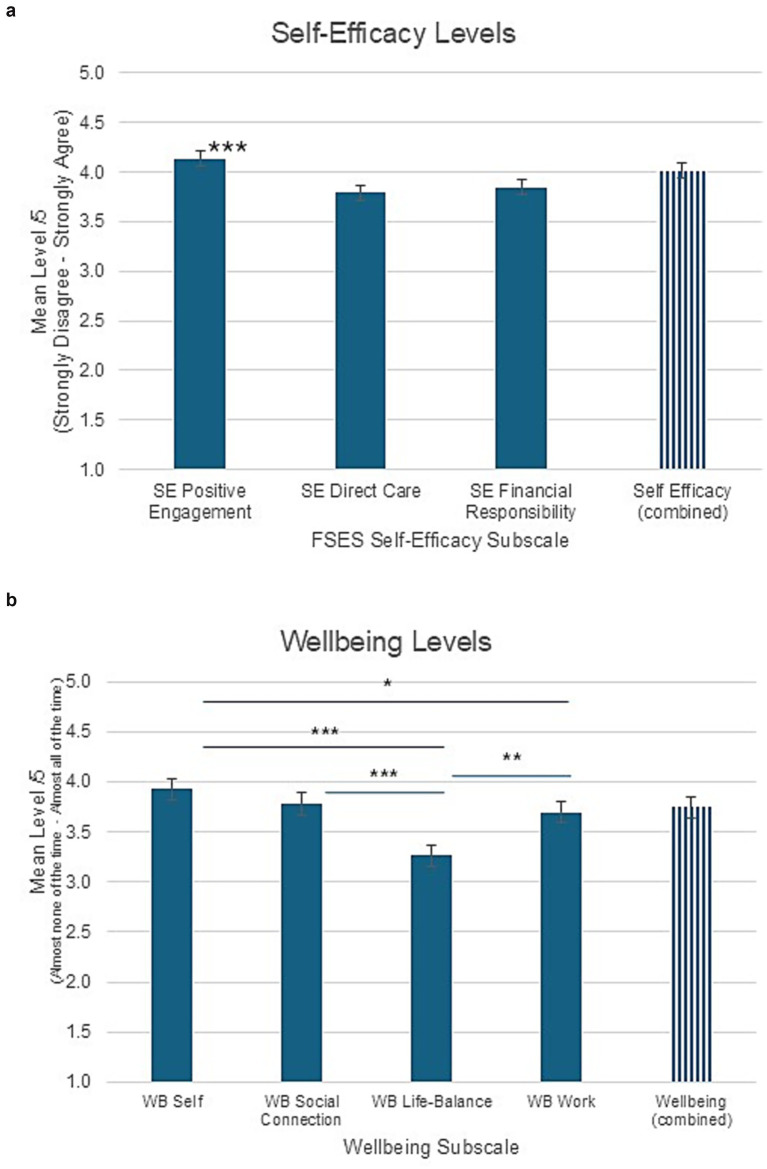
Levels of self-efficacy **(a)** on the Fathering Self-Efficacy Scale (Sevigny et al., 2016) and wellbeing **(b)** on the Being Well in Life and Work scale (developed here) for each of their subscales. Asterisks indicate significant differences with *p < 0.05, **p < 0.01, ***p < 0.001.

To evaluate fathering self-efficacy and its association with the wellbeing of fathers, The Fathering Self-Efficacy Scale (FSES) was used in the current context. It yielded excellent reliability (Cronbach’s *α* = 0.918). The repeated measures ANOVA indicated an effect across subscales [*F* (1.64, 191.86) = 15.3, *p* < 0.001], and means out of 5 ± SE for each of the three subscales are shown in Figure 2. Levels were highest for Positive Engagement (4.14 ± 0.59) compared to Direct Care (3.79 ± 0.58; *p* < 0.001) and Financial Responsibility (3.85 ± 0.84; *p* < 0.001); the last two were similar to each other. The total mean was 4.02 (±0.53).

### 3.4 Wellbeing – the being well in life and work scale (BWLW)

Data were factorable with numerous stronger intercorrelations > 0.3, and no evidence of multicollinearity (no bivariate *r*’s > 0.80). Bartlett’s test of sphericity was significant at p < 0.001, and the sampling size was good as indicated by Kaiser–Meyer–Olkin measure of sampling adequacy of 0.85. The Parallel Analysis indicated 3 factors explaining 46.9% of the variance, and the Scree plot (Cattell, 1966) revealed plausible solutions at 3 and 4 factors. Extractions were tested on these numbers of factors; the pattern matrix with the interpretation that was clearest with the framework, included four factors, and the final solution is shown in Table 3. Of the 26 original items, five did not load at the 0.40 threshold (items 6, 11, 16, 23, 24, 26), and items 2 and 21 did not fit their co-loading factor items, so all these items were dropped from the solution. The solution consisted of 18 items, loading onto four theoretically consistent factors and explaining 59% of the variance. Overall, model fit measures indicated a good fit with an RMSEA of 0.058 (CI 0.028–0.08; RMSEA is considered good at < 0.06 and adequate at <0.08), a TLI of 0.93 (adequate at >0.90 and good at >0.95); the chi square was significant [*χ*^2^ (87) = 118, *p* = 0.014] but the *χ*^2^/df ratio remained under 2, which is considered good (Hu and Bentler, 1999).

**Table 3:** Final items and factor loadings of the Being Well in Life and Work (BWLW) questionnaire after removal of low-loading (<0.4) and non-loading items (full scale items are shown in the Appendix Table 1).

#		Item		EFA loadings			
				Self in context	Social connectedness	Life balance	Work satisfaction
13	I feel confident in my own abilities	0.69			
12	I like facing challenges	0.68			
3	I have enough energy	0.63			
14	I feel comfortable in my daily activities	0.58			
15	I am sure I can do all that I need to do	0.55			
22	I am able to make my own decisions in life	0.46			
1	I am happy	0.43			
4	I get depressed (r)	0.41			
25	I prefer doing things with others		0.79		
19	I feel connected to my friends		0.78		
20	I enjoy being with others at work		0.59		
18	I find it easy to relate to other people		0.49		
9	I have enough time for myself			0.77	
8	I have a good work-life balance			0.73	
5	I am comfortable with my life as it is			0.45	
10	I look forward to going to work				0.72
7	I am satisfied with my career				0.71
17	I have a feeling of belonging at work				0.64

This scale may be used by citing this article; for translations, please contact the corresponding author.

Scale correlations and convergence. Bivariate correlations are shown in Table 4. Correlations between the wellbeing subscales (bottom-right triangle) yielded positive associations ranging between 0.43 and 0.67 (all *p* < 0.001), with strong correlations between each of the subscales and the overall wellbeing score (total: mean across all items) of 0.75–0.92 (all *p’s* < 0.001). Correlations conducted between the FSES subscales and the Wellbeing subscales yielded positive associations ranging from 0.36 to 0.64 (all *p’s* < 0.001).

**Table 4:** Correlation matrix for the fathering self-efficacy subscales, the wellbeing subscales, and between the FSES and wellbeing subscales.

Subscale	SE - PE	SE - DC	SE - FR	SE - total	WB - Self	WB - Social	WB - Life balance	WB - Work	WB - total
SE - Positive engagement	—								
SE - Direct care	0.60***	—							
SE - Financial responsibility	0.44***	0.39***	—						
SE – total	0.94***	0.73***	0.68***	—					
WB – self	0.64***	0.43***	0.41***	0.64***	—				
WB - Social connection	0.52***	0.41***	0.36***	0.55***	0.67***	—			
WB - life balance	0.48***	0.45***	0.41***	0.54***	0.60***	0.43***	—		
WB – work	0.49***	0.36***	0.38***	0.54***	0.61***	0.59***	0.52***	—	
WB - total	0.65***	0.49***	0.46***	0.68***	0.92***	0.82***	0.75***	0.80***	—

*p < 0.05, **p < 0.01, ***p < 0.001.

SE, Self-Efficacy scale (the FSES; Sevigny et al., 2016); with PE, Positive Engagement; DC, Direct Care; FR, Financial Responsibility; WB, Wellbeing scale (the BWBLW), Self, Social Connectedness, Life Balance, Work Satisfaction.

Wellbeing levels are shown in Figure 2. The repeated measures ANOVA indicated an effect across subscales [*F* (1.72, 325.86) = 16.8, *p* < 0.001] and means out of 5 ± SE for each of the four wellbeing subscales indicated fathers being well with oneself (3.92 ± 0.06) and their sense of social connection (3.78 ± 0.08) were similarly (*p* = 0.07) near a lot of the time. The sense of life balance was lower at about half the time (3.26 ± 0.09, all *p*’s < 0.001). Work wellness (3.70 ± 0.09) was between about half the time and a lot of the time; its level was similar to that of social connection, lower than the sense of self-wellness (*p* = 0.02), but higher than life-balance (*p* = 0.004). The mean for wellbeing overall was 3.74 ± 0.06.

### 3.5 Positive engagement, self-efficacy, and wellbeing

To assess whether (1) self-efficacy and (2) wellbeing could predict either positive engagement literacy activities (total score) or reading with children, generalized linear model analyses indicate that self-efficacy but not wellbeing were associated. Specifically, self-efficacy on the FSES was supportive of the positive-engagement literacy score, with the model explaining 7.5% of the variance (*R*^2^ = 0.0754), through Financial Responsibility χ^2^(1) = 5.77, *p* = 0.016, but not Positive Engagement (*p* = 0.28) or Direct Care (*p* = 0.07). Parameter estimates indicate that Financial Responsibility had a positive effect on engagement in literacy development, B = 0.28, SE = 0.12, z = 2.40, *p* = 0.018, 95% CI [0.12, 0.45], whereas Positive Engagement and Direct Care were not (PE: B = 0.21, SE = 0.19, *p* = 0.281; DC: B = −0.36, SE = 0.20, *p* = 0.073). The same pattern of predictors was found for reading paper books or magazines with children, with the model explaining 8.1% of the variance (R^2^ = 0.081) through Financial Responsibility χ^2^(1) = 4.20, *p* = 0.04, but not through Positive Engagement (*p* = 0.11) or Direct Care (*p* = 0.26). Financial Responsibility had a positive effect on engagement in literacy development, B = 0.34, SE = 0.16, z = 2.05, *p* = 0.043, 95% CI [0.2, 0.65], whereas Positive Engagement and Direct Care did not (all *p* > 0.1). Wellbeing was not predictive of engagement in literacy activities (total score) or in reading with children (all *p*’s > 0.1).

Self-efficacy subscales of the FSES predicted wellbeing with the model explaining 46.2% of the variance (*R*^2^ = 0.462) through Positive Engagement [*χ*^2^(1) = 30.74, *p* < 0.001] and Financial Responsibility [*χ*^2^(1) = 6.90, *p* = 0.009], but not Direct Care (*p* > 0.1). Parameter estimate effects on wellbeing were positive for the FSES’ Positive Engagement (B = 0.60, SE = 0.11, z = 5.54, *p* < 0.001, 95% CI [0.39, 0.80]) and Financial Responsibility (B = 0.17, SE = 0.07, z = 2.63, *p* = 0.010, 95% CI [0.04, 0.29]).

## 4 Discussion

The purpose of this study was to address the wellbeing of fathers through their engagement in literacy activities with their children, their beliefs about reading, and their fathering self-efficacy, and to develop a wellbeing tool. The wellbeing tool, Being Well in Life and Work, incorporated cognitive and affective satisfaction (Diener et al., 1999, 2018) of the three core psychological needs, in terms of one’s sense of competence, autonomy, and belonging (Ryan and Deci, 2000, 2017) across aspects of life and work that are relevant to fathers as individuals. The fathering self-efficacy scale (FSES) consists of three subscales: *Positive Engagement* with children, *Direct Care* of children, and indirect care through *Financial Responsibility* (Sevigny et al., 2016).

### 4.1 Wellbeing questionnaire – being well in life and work (BWLW)

One of the subgoals of the present study was to evaluate a wellbeing questionnaire relevant to fathers as individuals. Results from the Exploratory Factor Analyses (EFA) yielded a four-factor structure across 18 items: a sense of *Self*, a sense of *Social Connectedness*, a sense of *Life-Balance*, and a sense of *Work Satisfaction*. The sense of Self incorporated items related to personal feelings including affective understanding (except for #2 “I easily get stressed”), items underlain by competence (except #16 “I find it easy to complete my work”), and an item based on autonomy regarding being able to make our own decisions in life. This suggests that for fathers, overall affect, competence, and the ability to make life decisions (autonomy) come together in their sense of self, and that the satisfaction of competence is prominent for father wellbeing. This is consistent with the associations between wellbeing and fathering self-efficacy in the present work, and in previous findings of father self-efficacy and the satisfaction of basic psychological needs (Bouchard et al., 2007; Fortin, 2023). Congruently, the items addressing stress and work completion did not load, likely because of their situational occurrence (i.e., stress is frequently elicited by external factors, as are work tasks), and while external factors, including stress, can affect wellbeing (e.g., Wong et al., 2021), they do not seem associated with fathers’ personal feelings of themselves. Having a balanced life (Life-Balance) was associated with cognitive aspects of wellbeing involving autonomy fulfillment, such as comfort with life, having enough time for oneself, and feelings of work-life balance. This autonomy-related pattern was interesting, because, of the other items more directly associated with autonomy, only two loaded onto other parts of the scale. The pattern of one item loading “I prefer doing things with others” with another not loading “I prefer to do things independently” (item 26), could have been associated with dependence and lesser autonomy. However, the interpretation is more likely related to feelings of connection with others (relatedness), reflecting the local culture of knowledge sharing (Rao et al., 2022) and moderate collectivism in the UAE (Pelham et al., 2022). The other two autonomy-associated items pertaining to decisions about activities in daily life and at work, did not load, which is coherent with the items on stress and work-completion not loading; fathers likely view these elements to be external to their understandings of themselves or their sense of balance. The pattern of loadings for Social Connectedness involved relationships with others across life and work, consistent with the importance of social support to the wellbeing of fathers (e.g., Nelson et al., 2014) and of warm relationships to the satisfaction of psychological needs (Deci and Ryan, 1985, 2000; Ryan and Deci, 2000, 2017). However, somewhat surprisingly, feeling close to one’s family did not load; closeness with family can involve feeling connected, but can also carry a sense of responsibility or duty through fathers’ sense of family provision (Sevigny et al., 2016), and accordingly, this item was not associated with the other items of the construct, which carried stronger links to mutual relationships. Work Satisfaction encompassed items associated with cognitive evaluation such as career satisfaction, and with a sense of belonging at work, consistent with previous findings of wellbeing at work (Olafsen et al., 2021). Confirmatory factor analysis could not be run due to sample limitations, but the structure obtained here was consistent with the fathering self-efficacy scale – FSES (convergent validity) and explained almost 60% of the variance (ideals are between 60% and 70%). Considering the extensive cultural diversity across the numerous nationalities and backgrounds in this sample, with varying cultures and contexts, this percentage of explained variance is relatively robust. The structure of the questionnaire needs to be reaffirmed through confirmatory factor analysis with larger samples and across other contexts, but the preliminary structure is likely to apply more widely given the cultural diversity of the present sample, with varieties of English, Arabic, and other languages. This is consistent with previous reports that satisfaction of the three core psychological needs feeds into positive functioning across dimensions of wellbeing and cultures (e.g., Chen et al., 2015, Disabato et al., 2016; Martela and Sheldon, 2019). In addition, an analysis of wellbeing in the UAE defined through life satisfaction (based on Gallup data spanning 10 years), showed an overall life satisfaction evaluation of 70% of their scale (Lambert et al., 2020), which is comparable to the overall wellbeing of fathers in the present study at 75% of our BWLW scale.

### 4.2 Father wellbeing and self-efficacy

As father wellbeing can be associated with fathering self-efficacy, these were evaluated, and findings indicate that the overall wellbeing of fathers and their fathering self-efficacy were relatively high. Fathering self-efficacy was highest for positive engagement activities with children, followed by slightly lower levels of direct and indirect care via financial responsibility. In terms of wellbeing, perceived self-wellness and social connectedness were highest, with slightly lower work wellness, and the sense of life balance was lowest. The wellbeing of fathers was supported by fathering self-efficacy, mainly by Positive Engagement and to a lesser extent by Financial Responsibility, but not by self-efficacy in Direct Care of children.

This is consistent with previous work indicating that increased involvement in direct care and household chores could be associated with decreased psychological health (Blair and Hardesty, 1994), whereas aspects of fulfillment of indirect care, such as financial responsibility, are important to fathers (Pleck, 2010; Sevigny et al., 2016). Findings from large-scale analyses of the wellbeing of individuals in the region, shows wellbeing associated with income and with positive engagements in life, such as donating (Lambert et al., 2020), which can be considered a form of indirect care (to society), and which suggests that fathers may value indirect care for their children and more widely. In terms of aspects of wellbeing, the perceived sense of self-wellness and social connectedness were highest for fathers, with slightly lower work wellness, and the lowest was the sense of life-balance, which comprised items such as “I have enough time for myself”. Finding that the sense of social connection in wellbeing was high, is also consistent with the large-scale work in the local context (Lambert et al., 2020). For fathers, work on the benefits of social support for father psychological wellness (Nelson et al., 2014) and for father self-efficacy and involvement with children (Bakermans-Kranenburg, 2022; Fortin, 2023), has focused on social support from the co-parent, but we highlight here that a sense of social connectedness through reciprocal relationships with friends, work, and general relatedness, seems to contribute to father wellbeing. Supporting fathers with what is relevant to them is consistent with fathers expressing the need for father-specific support (Gaynor et al., 2024) and from the lasting benefits of father-targeted training (Hearn et al., 2020; Clarkson and Hearn, 2021).

### 4.3 Literacy engagement, father wellbeing and self-efficacy

#### 4.3.1 Literacy engagement

In addressing the contribution of fathering self-efficacy and wellbeing to literacy development, father engagement in literacy development activities were examined. The frequency of engagement in literacy-supporting activities was relatively low, averaging a few times per month, and yet the range was broad, as it was for beliefs, suggesting a large diversity in home reading practices. This broad range of engagement frequency in literacy practices could be due to a variety of factors beyond the current scope, but one aspect to consider is that fathers appreciate resources suited to them (Gaynor et al., 2024). For example, our previous findings on shared-reading workshops with fathers indicated that fathers frequently did not know what to read with their children and appreciated relevant suggestions for children’s books, as well as father-specific shared reading interactions (Gallagher et al., 2025). In addition, in the present sample, three items did not load on the scale assessing engagement in activities that support literacy development. One item addressed reading on electronic devices, one addressed the frequency of play, and the last addressed fathers’ enjoyment of reading with their child. The item on reading *paper* books or magazines loaded, but not reading books or magazines on devices, suggesting that fathers may not view devices as associated with reading. This is consistent with recent findings that many parents who engage in shared reading with their young children do not do so digitally because of concerns about screen time limits, and because they associate screen time with children’s unsupervised device use; father–mother comparisons were not conducted (Nicholas and Paatsch, 2024). In the present sample, fathers reported engaging in play with their children regularly (more than several times per week), which was significantly higher than engagement in any of the literacy activities, but play did not load with these activities. It appears that play is not understood as contributing to literacy development among fathers in this sample, similar to mothers in the UAE (no known work on fathers) not viewing play as associated with learning (Al-Qinneh and Abu-Ayyash, 2022), even though play is valued by parents in the region (Foulds, 2023), including the fathers here, who reported frequently playing with their children. This suggests that father-targeted resources and interventions should include elements on the nature and depth of play, and its importance for children’s learning and development. In addition, given the relatively low frequency of engagement in literacy-supporting activities, father interventions could incorporate these activities into father-child play. Children’s enjoyment loaded on the scale, while fathers’ own enjoyment did not, even though they were rated similarly. A plausible explanation is that fathers do not view their own enjoyment as associated with their children’s literacy development, and that engagement in literacy-supporting activities may be something they do *for* their child’s learning rather than meaning-making *with* their child. This echoes findings from fathers participating in shared reading workshops, in which fathers viewed shared reading as an advantage for language development, positioning it as an investment in their children rather than leisure (Gallagher et al., 2025). This does not mean that fathers do not value enjoyment with their children, findings here and elsewhere indicate that they do (e.g., Swain et al., 2017; Dillon et al., 2024; Gallagher et al., 2025) but rather, that they do not associate their own enjoyment with their children’s learning or literacy development.

#### 4.3.2 Literacy engagement and father self-efficacy

Father engagement in activities that support literacy development, including reading paper books, was predicted by self-efficacy through Financial Responsibility, but not by any aspects of wellbeing. This pattern appears to reflect that financial provision is a central aspect of paternal responsibility (or dimension of father commitment) across many cultural groups in the UAE, including Emirati and expatriate fathers working and residing in the country. As a result, perceived financial responsibility and capability of fathers serve as a proxy for breadwinner identity or socioeconomic status, which enables the provision of literacy resources and structured learning opportunities (= literacy engagement). Drawing on father responses and engagement during workshops (Gallagher et al., 2025), reading with children seems to be a social investment in children’s future success. This idea is consistent with fathers considering their own enjoyment separate from literacy engagement with their child, which could also explain our finding that father wellbeing did not predict literacy engagement. However, in our previous findings, fathers who participated in shared-reading workshops described positive experiences, such as enjoyment, bonding, and closeness during shared reading (Gallagher et al., 2025), which continued during shared reading at home (Dillon et al., 2024). In addition, the present work’s correlation between Positive Engagement (FSES) and father wellbeing indicates that relational and affective qualities, such as those of shared book reading are associated with father wellbeing. The pattern of wellbeing not predicting literacy engagement could arise from fathers’ infrequent engagement in activities that support children’s literacy development (a few times per month), which was not sufficient to support a connection, especially given the concurrent broad range of engagement frequency. Together, these facets suggest that the infrequent engagement of fathers in activities supportive of literacy development can be addressed through specific father-targeted interventions tied to play, as fathers reported playing with their children more than several times per week. In addition, the levels of education and employment were relatively high in the present sample, which could limit our findings; however, most items pertaining to language and literacy development displayed a very broad range of engagement practices and beliefs. The range of beliefs was so wide, that none were associated with any aspect of self-efficacy. This suggests that, despite limitations arising from most participants being employed or well educated, the extensive cultural and linguistic diversity may allow for greater relevance to other contexts.

The effect of self-efficacy on literacy engagement was small, so other factors not included in the model, such as father-relevant resources or family practice diversity as discussed above, could play a role. However, the finding that self-efficacy in Financial Responsibility was significant in predicting literacy engagement is meaningful, because it suggests that a father’s sense of family provision (a form of indirect care) is supportive of direct engagement, here literacy-supporting activities that are beneficial to the child. This is consistent with indirect care being important to fathers (Pleck, 2010; Sevigny et al., 2016), as is the still-relevant sense of provision (Lamb, 2000; Christiansen and Palkovitz, 2001) despite a shift towards more involved father practices (Dermott and Miller, 2015), and supports the notion that fulfillment of the psychological needs of fathers (here self-efficacy) contributes to their involvement (Bouchard et al., 2007; Fortin, 2023). Interestingly, fathers’ perceived energy in engaging with their children’s schooling is associated with improvements in their self-efficacy, regardless of long working hours, which are usually considered a barrier (Bi et al., 2021). Together with our findings, this suggests that a father’s perceived self-efficacy, through indirect care, can influence the quality of father-child literacy interactions and development, with wider implications for considering work environments that are supportive of fathers.

Interestingly, while beliefs about literacy were not associated with father engagement in literacy development activities, father self-efficacy, nor father wellbeing, the concurrent notion of fathers seeming to engage “for” their child rather than “with”, points to a plausible need for fathers to develop a deeper, intentional, appreciation of their child’s thinking or mind (Mind-Mindedness^3^; Meins, 1997, Meins, 2013). This suggestion is further supported by the father-perceived dissociation between their own enjoyment and that of their children when reading; even though fathers are sensitive to their child’s internal state (recognizing enjoyment), they do not seem to connect it to their own, perhaps because of a focus on child needs rather on the child as an individual (Meins, 1997, Meins, 2013) with sophisticated thinking. However, given our previous findings that after participation in a shared reading workshop, fathers reading with their child did describe enjoyment, bonding, and closeness (Dillon et al., 2024; Gallagher et al., 2025), the present work’s association between the positive engagement of fathers and their wellbeing, and the frequency of father engagement in play, suggests that shared reading and play, could be a means by which fathers can be intentionally (Dennett, 1989; Meins, 2013) attuned with their child.

## 5 Conclusion

The integration of sophisticated aspects of fathering, including father wellbeing and its interaction with children, suggests that reading with children is a positive interaction that could benefit both the father and the child. Of particular interest are the importance fathers place on indirect care, its association with direct engagement in literacy-supporting activities, and the benefits of father-specific resources, suggesting the necessity of scaling parenting interventions to target fathers specifically with approaches relevant to them, such as incorporating play with literacy. Finally, in considering the wellbeing of fathers themselves, opportunities for self-efficacy through social connectedness and positive engagement can support their wellbeing.

## Data Availability

The raw data supporting the conclusions of this article will be made available by the authors, without undue reservation.

## Footnotes

1
https://www.qualtrics.com

2
https://www.jamovi.org/

3 We would like to thank a reviewer for suggesting to look into mind-mindedness in this context.

## Appendix

**Table A1:** Initial scale items and their connection to dimensions of wellbeing according to the three psychological needs (feelings of competence, autonomy, and relatedness) (Deci and Ryan, 1985; Ryan and Deci, 2000, 2017) and life satisfaction in terms of affective-cognitive tone (Diener et al., 2018, 1999) are indicated in the last column.

	Item	Category	Main psychological need and satisfaction (affective/cognitive)
1	I am happy	Self	Affective
2	I easily get stressed (*r*)	Self	
3	I have enough energy	Self	
4	I get depressed (*r*)	Self	
5	I am comfortable with my life as it is	Life	Cognitive (autonomy)
6	I am satisfied with my life	Life	
7	I am satisfied with my career	Work	
8	I have a good work-life balance	Life	
9	I have enough time for myself	Life	
10	I look forward to going to work	Work	
11	I look forward to going home	Home	
12	I like facing challenges	Self	Competence
13	I feel confident in my own abilities	Self	
14	I feel comfortable in my daily activities	Self	
15	I am sure I can do all that I need to do	Self	
16	I find it easy to complete my work	Work	
17	I have a feeling of belonging at work	Social/Work	Relatedness
18	I find it easy to relate to other people	Social	
19	I feel connected to my friends	Social	
20	I enjoy being with others at work	Social	
21	I feel close to my family	Social	
22	I am able to make my own decisions in life	Self	Autonomy
23	I am able to make decisions about my daily activities	Life	
24	I like being able to make my own decisions at work	Work	
25	I prefer doing things with others	Social	
26	I prefer doing things independently	Social	

The categories involving aspects relevant to life and work are indicated in the before-to-last column.
